# Sorption properties of several polyethersulfone membranes for hydrophilic and hydrophobic emerging contaminants: implications for their application in passive sampling

**DOI:** 10.1007/s00216-026-06476-4

**Published:** 2026-04-20

**Authors:** Chiara Scapuzzi, Henry MacKeown, Barbara Benedetti, Marina Di Carro, Emanuele Magi

**Affiliations:** Department of Chemistry and Industrial Chemistry, University of Genoa, Via Dodecaneso 31, 16146 Genoa, Italy

**Keywords:** Polymer characterization, Membrane-water partition coefficients, Passive sampling, Aqueous matrices, Principal component analysis

## Abstract

The presence of emerging contaminants (ECs) in aqueous matrices has gained attention during the last decades due to their possible negative effects on the environment and on human health. In this context, passive sampling represents a promising strategy for the monitoring of ECs in water. Polyethersulfone (PES) membranes have been employed in passive samplers both as a sorptive material and as a protective layer. To understand the properties of PES in the sorption of ECs with different physico-chemical properties and develop a reliable sampling strategy, PES-water partition coefficients (*K*_PESw_) can be evaluated. In the present work, *K*_PESw_ was investigated for 37 compounds using PES membranes with the same pore sizes (0.1 μm) but coming from different suppliers and a PES membrane with a larger pore size (0.45 μm). *K*_PESw_ values were compared based on pore dimensions and supplier, and different sorption trends were observed. Moreover, two different setups were employed to evaluate the partition coefficients using the most employed PES membranes in passive sampling applications. The log*K*_PESw_ values obtained, ranging from −0.1 ± 0.2 to 6.26 ± 0.03, were correlated with the analytes’ physico-chemical properties using a multivariate approach, highlighting hydrophobicity as the main driving force in the uptake. Finally, the *K*_PESw_ values obtained in this work and in previous studies were correlated with the analyte’s distribution coefficients, confirming the influence of the hydrophobic nature of the compounds on the sorption. These observations can guide the design of monitoring campaigns, providing hints on the proper material to use for sampling and on the selection of target compounds.

**Graphical abstract:**

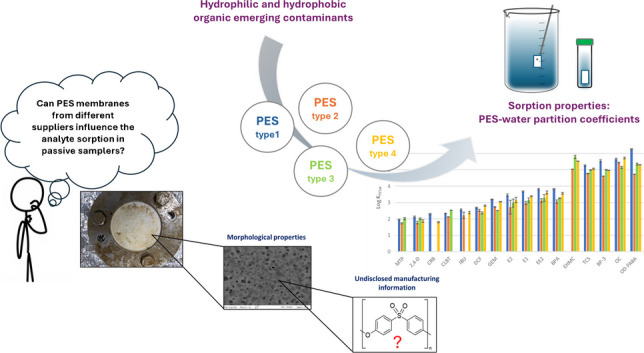

**Supplementary Information:**

The online version contains supplementary material available at 10.1007/s00216-026-06476-4.

## Introduction

Passive sampling (PS) represents a promising strategy for the monitoring of organic emerging contaminants (ECs) in water matrices, allowing the assessment of EC concentration over extended periods of time but limiting the laboratory efforts required. Several devices have been developed to detect ECs with different physico-chemical properties, mainly in two configurations: single-phase (sorbent) and dual-phase (sorbent and protective layer) samplers.

Partition coefficients between the PS material and the sample medium represent an important parameter to evaluate, as they provide information on the affinity of a potential receiving phase for the target compounds. Moreover, they are fundamental for equilibrium passive samplers, since the accuracy of the partition coefficient of a specific compound strongly affects the accuracy in the estimation of its water concentration (C_w_) [1]. The evaluation of these coefficients is also important for passive samplers employed in the integrative uptake regime. However, very few studies have measured the partition coefficient of the protective layer in dual-phase passive samplers [2]. Nonetheless, these measurements can be useful to better understand the uptake of chemicals by the sampling device [3–5]. In fact, sorption onto/into the protective membrane could affect the uptake kinetics of the receiving phase, causing lag times and, as a consequence, lowering the sampling rates of some compounds or affecting the linearity of the uptake. In addition, some analytes which tend to sorb strongly to the membrane could be excluded from the application of the dual-phase passive sampler [5].

The polymer-water partition coefficient can be determined by allowing a chemical to reach equilibrium in its distribution between water and polymer, followed by analysis of both phases. To reach equilibrium, aqueous concentrations may be maintained constant (constant C_w_ design) or allowed to change over time (single-dose design) [1]. In the constant C_w_ design, changes in the water concentration of the analyte are kept negligible by using flow-through systems or dosing materials [6]. The single-dose method consists of a sole spike of analytes into the water, which is then no further renewed. In both methods, the aqueous phase concentrations used are often very low and difficult to measure accurately, particularly for the more hydrophobic compounds that may sorb appreciably to the walls of the container or to dissolved organic matter present in the water. To avoid these issues, partition coefficients could be measured in water-miscible cosolvent systems, to then extrapolate the values in the case of zero cosolvent, or three-phase systems by adding a micelle forming surfactant to reduce time to equilibrium [7].

Polyethersulfone (PES) has been used as a single-phase sampler for the detection of ECs in different aqueous matrices [8] and is the typical protective layer of the most employed dual-phase samplers, the polar organic chemical integrative sampler (POCIS) and the Chemcatchers [2]. Experimental PES-water partition coefficient (K_PESw_) values have been obtained for a variety of nonpolar and polar compounds. Some of these values were measured using the single-dose method [4, 9, 10] or the constant C_w_ design [11] as part of POCIS and Chemcatchers. Regarding the PES membrane as a single-phase passive sampler, the single-dose method was mainly employed to estimate the K_PESw_ [5, 12–15], while only Chepchirchir et al. (2020) performed a constant C_w_ design [16]. Instead, Estoppey et al. (2019) calculated K_PESw_ fitting experimental data with a two-compartment model [17]. A recent article collected LogK_PESw_ values for 125 compounds from 9 studies either directly from the literature or calculated from graphical data using image processing features [5]. However, these K_PESw_ have not always been evaluated at the equilibrium, impacting the accuracy of their values [8]. Another important parameter to consider when investigating the sorption properties of PES is the membrane pore size. In POCIS application PES membranes of 0.1 μm pore size are usually employed as a protective layer; however, for some compounds (in particular, hydrophobic compounds), the sorption on PES, thus the lag time, can be reduced using membranes with larger pores (e.g., 5 μm pore size) [18]. Also, Kaserzon et al. (2014) observed increased sampling rates for polar and ionizable compounds comparing PES membranes of 0.2 μm and 0.45 μm using Chemcatchers™ samplers [10]. In the latter case, membranes of different pore sizes may present differences in porosity and tortuosity, influencing the mass transfer resistance through the protective layer when diffusion occurs through the water filled pores. These findings can be addressed to the different diffusion mechanisms of the analytes: through the water filled pores or through the polymer matrix [19].

Only one study has investigated the use of PES polymer as a passive sampler in different formats (PES tubes or PES membranes), so far [20]. However, no study has yet considered the possible differences in analyte sorption to PES membranes from different suppliers due to differences in their non-disclosed manufacturing processes. Analogously, for example, some very large differences in affinity for organic compounds were found between two kinds of silicone rubber samplers [21–23].

In the present work, the typical flat sheet PES membranes of 0.1 μm pore size used in POCIS applications, provided by three different suppliers, were tested. In addition, a PES membrane of 0.45 μm pore size was studied to evaluate the influence of pore dimensions on sorption. The partition coefficients of several ECs covering a broad polarity range were evaluated to assess the possible impacts on the POCIS uptake. Furthermore, the investigated membranes were characterized in terms of morphology, functional groups, and porosity to improve understanding on the sorption mechanisms.

## Materials and methods

### Chemicals and reagents

Ultra-pure water, methanol (MeOH), and acetonitrile (ACN) were purchased from VWR (Radnor, PA, USA). Acetic acid (CH_3_COOH, “AA”) was provided by Sigma-Aldrich (St. Louis, MO, USA). All solvents were HPLC-MS grade. Water employed for the evaluation of PES-water partition coefficients was purified by a Milli-Q system (Millipore, Watford, Hertfordshire, UK).

Several emerging contaminants were investigated including UV filters, estrogens, pharmaceuticals and personal care products, and more hydrophilic pesticides. Analytical standard solutions were prepared by dissolving or diluting in MeOH or MeOH:water 1:1 (v/v) pure powders, liquid standards and certified grade solutions (all above 98% of purity) purchased from different suppliers: theophylline (THEOP), theobromine (TBR), carbamazepine (CBZ), benzophenone-3 (BP-3), octyl dimethyl p-aminobenzoate (OD-PABA), ethyl hexyl methoxy cinnamate (EHMC), octocrylene (OC), perfluorooctanoic acid (PFOA), acesulfame (ACS), sucralose (SCL), bisphenol A (BPA), estrone (E1), β-estradiol (E2), 17α-ethinyl estradiol (EE2), ibuprofen (IBU), gemfibrozil (GEM), clenbuterol (CLBT), hydrochlorothiazide (HCTZ), furosemide (FRSM), and triclosan (TCS) from Sigma-Aldrich (St. Louis, MO, USA); caffeine (CAFF), ketoprofen (KET), naproxen (NAP), and diclofenac (DCF) from Fluka Analytical (Saint Gallen, Switzerland); salbutamol (SLBT) from Alfa Aesar (Haverhill, MA, USA); taurine (TAU), omethoate (OMT), daminozide (DMNZ), 2,4-dichlorophenoxyacetic acid (2,4-D), chloramphenicol (CMPH), metformin (MTF), atenolol (ATN), terbutaline (TRBT), chlormequat (CMQ), fluroxypyr (FXP), and metoprolol (MTP) from Merck (Darmstadt, Germany).

Table S1 summarizes the main physico-chemical properties of the target compounds.

### Polyethersulfone membranes

#### Selection

Microporous membranes, in the form of rectangular sheets, with 0.1 μm pore size were obtained from Pall Italia (P01) (Buccinasco, Italy). The other two membranes were in the form of disk filters of 90-mm diameter and 0.1 μm pore size, from Hangzhou Anow Microfiltration Co., Ltd (H01) and Sterlitech Corporation (S01) (WA, USA). PES membrane disk filters of 0.45 μm pore size (P045)—which is the typical pore size employed in diffusive gradients in thin films (o-DGTs) devices—were from Pall Italia (Buccinasco, Italy).

#### Pre-wash considerations

Polymer pre-extraction methods should be documented because the residual oligomer content may influence the partition coefficient [1]. As the H01 and S01 membranes came from commercial POCIS, thus already pre-assembled, no additional pre-wash to any already performed by the POCIS supplier was carried out. On the contrary, P01 and P045 were pre-washed before assembly in the laboratory as previously described [18].

#### Membrane characterization

PES membranes in dry state were characterized in terms of microscopic surface morphology. A field emission scanning electron microscope (FE‐SEM, ZEISS SUPRA 40 VP (White Plains, NY, USA)) was used to obtain images at different magnifications (125×–30,000×) of the outer and inner surfaces and of the cross-section. The membranes were coated by a thin layer of graphitic carbon to render them conductive.

Attenuated total reflectance Fourier transform infrared (ATR-FTIR) spectroscopy was employed to assess the presence of different functional groups in the polymer due to different manufacturing processes. A Spectrum 65 FT-IR spectrometer (PerkinElmer, Waltham, MA, USA) equipped with a KBr beamsplitter, a DTGS detector, and a diamond crystal ATR accessory was used. The spectra were recorded in the wave number range from 4000 to 600 cm^−1^.

A gravimetric method was employed to calculate the porosity (P) of the different PES membranes [24]. Membrane sheets (three replicates) were cut into pieces of known area and weighed. The thickness of the sheets was measured with a micrometer. The membranes were immersed in ultra-pure water for 20 h. Afterwards, the water on the surface was carefully removed and the membranes were weighed. Porosity (P) was thus calculated as an averaged value by the following equation (Eq. 1):

1 $$\begin{array}{c}P=\frac{w_{w\;}-\;w_d}{A\;\cdot\;\delta\;\cdot\;d_w}\end{array}$$

where *w*_*w*_ is the weight of the wet membrane, *w*_*d*_ the weight of the dry membrane, *A* the membrane area, *δ* the thickness of the membrane sheet, and *d*_*w*_ the water density (0.997 g cm^−3^).

The contact angle of PES membranes was measured using the sessile drop method with an Attension® contact angle meter (NanoScience Instruments—Phoenix, AZ, USA).

#### Membrane processing

After batch experiments (see section “Batch experiments”), the analytes were extracted from the PES membrane by the following protocol: 12 mL of MeOH were added into a 20-mL vial containing the membranes, and the extraction was performed on a rotator drive (STR4/4, Stuart, UK) at 36 rpm for 30 min. The eluate was transferred to a flask, and the procedure was performed twice. The eluate was then reduced to dryness on a rotary evaporator (Rotavapor® R-100, BUCHI, Switzerland) and reconstituted in 1 mL of methanol; this solution was filtered through a 0.2-µm hydrophilic PTFE filter.

Blank membranes were extracted to assess possible sources of contamination during the sample treatments.

#### Recovery, matrix effects, and instrumental verifications

The reliability of the analytical evaluations was verified through several quality control strategies. Accuracy of the analytes’ determination in the PES was estimated as recovery and matrix effects. PES membranes of 6 cm^2^ (total surface area: 1.5 cm × 2 cm) were wetted with Milli-Q water and spiked with 25 µL of a 2 mg L^−1^ mix standard solution in MeOH. Once dry, the membranes were rinsed in a beaker containing 2 mL of Milli-Q water, left to dry again, and extracted as described in section “Membrane processing”. The final extracts were diluted 5-fold (spike before extraction: sample B), reaching a final concentration of 10 μg L^−1^ in the case of 100% theoretical recovery.

Blank membranes were extracted using the same procedure, 5-fold diluted and spiked to obtain a final concentration of 10 μg L^−1^ (spike after extraction: sample A).

Recovery (R%) was calculated by the following formula:

2 $$\begin{array}{c}R\%=100\;\frac{A_{B\;}-\;A_{NS}}{A_A\;-\;A_{NS}}\end{array}$$

where *A*_*B*_ and *A*_*A*_ are the LC-MS peak areas obtained by analyzing samples B and A, respectively. *A*_*NS*_ corresponded to the signal in the blank membrane extract.

Moreover, the Milli-Q water employed to rinse the membrane was analyzed after dilution with MeOH (1:1, v/v) to evaluate the loss in water of not sorbed compounds.

Alongside recovery, the possible matrix effect observed during LC-MS analysis of the extracts was evaluated and calculated using the following expression:

3 $$\begin{array}{c}ME\%=100\;\frac{A_{A\;}-\;A_{NS}}{A_{std}}\end{array}$$

where *A*_*A*_ is the LC-MS peak areas obtained by analyzing sample A, while *A*_*NS*_ and *A*_*std*_ are the peak areas obtained by analyzing the blank membrane extract and a neat standard at 10 μg L^−1^, respectively.

Other operations to ensure reliability of the analytical results were analysis of all samples obtained with the same setup in the same batch; replicated injections; calibration curve included in each batch to guarantee the linear relationship between concentration and peak areas in the considered concentration ranges; and analysis of several blanks and standards in different points of the LC-MS worklist to assess possible carryover and changes in the instrumental response during time.

### Batch experiments

#### Single-dose design

The evaluation of PES-water partition coefficients is usually carried out by the single-dose design [8]. Using this method, the analytes’ concentration in the water phase changes with time: an initial spike of the analytes is performed and the K_PESw_ is estimated by the amount of the target compounds sorbed onto/into the receiving phase compared to the final analytes’ concentration in water, when equilibrium is attained (Eq. 1) [1].

4 $$\begin{array}{c}{\mathrm{K}}_\mathrm{PESw}=\frac{C_{PES,eq}}{C_{w,eq}}\end{array}$$

The time to reach equilibrium (*t*_*eq*_) is proportional to the water volume and inversely proportional to the polymer mass. However, for high K_PESw_ values, the total amount of compound spiked into the selected water volume must be carefully chosen to avoid exhaustive extractions. Thus, considering the poor solubility in water of some of the more hydrophobic compounds and their K_PESw_ values presented in a previous work [14], a concentration of 10 μg L^−1^ in 2 L of Milli-Q water for all the target compounds and PES membranes of 6 cm^2^ (corresponding to an averaged weight of 9.8 ± 0.8 mg for H01, 13.3 ± 0.3 mg for P01, and 12.8 ± 0.2 mg for S01) was selected (S1 setup). This concentration also permitted obtaining final aqueous concentrations above the limits of quantitation [1]. PES membranes were exposed in beakers filled with spiked water. In each beaker, only one PES membrane was deployed using a steel wire. A water temperature of 25 °C and dark conditions were maintained during the experiment to obtain more accurate partition coefficients (which are temperature dependent) and to avoid the photodegradation of the more photosensitive compounds. The solution was stirred using magnetic stir bars and F30 magnetic stirrers (Falc Instruments, Italy). The incubation time was 14 days; several aliquots of water were withdrawn to monitor changes in the aqueous concentrations. This experimental configuration was applied to evaluate the K_PESw_ of the four PES membranes mentioned in section “Selection.” Two replicates were performed for each type of membrane, and a spiked solution without any receiving PES phase was subjected to the same experimental conditions to assess the potential loss of the analytes during the exposure time (e.g., degradation and/or sorption onto magnetic stir bars/glass walls).

To assess the partition coefficients also for those compounds with poor affinity for the PES membrane, a second single-dose setup was employed (S2 setup). Two vials with 20 mL of Milli-Q water were spiked to obtain two different concentration levels: 10 μg L^−1^ for BPA, estrogens, TCS and the four UV filters, and 100 μg L^−1^ for the other analytes. PES membranes were added to the vials, and an incubation of 21 days was performed. To monitor the analytes’ concentration in water during the exposure, 50 μL of solution was withdrawn at the beginning, in the middle, and at the end of the deployment.

After the exposure experiment, the dry PES membranes were extracted as described in section “Membrane processing.”

#### Cosolvent method

Methanol was added to water obtaining mixtures at 0%, 10%, 20%, 30%, and 40% (v/v). Afterwards, the target compounds were spiked into two vials with 20 mL of each solution to achieve two different concentration levels. These levels were selected considering the expected affinity for the receiving phase, the analytes’ solubility, and the sensitivity of the instrumental analytical method: 10 μg L^−1^ for BPA, the three estrogens, TCS and the four UV filters, and 100 μg L^−1^ for the other analytes. PES membranes were added to the vials, and an incubation of 21 days was performed, with 50 μL of water withdrawn at the beginning, in the middle, and at the end of the deployment.

After the exposure, the dried PES membranes were extracted as described in section “Membrane processing.”

### Instrumental analysis

A 1200 series HPLC coupled to a 6430 triple quadrupole mass spectrometer by Agilent technologies (Santa Clara, CA, USA) was employed for the analysis. The liquid chromatograph was equipped with a binary pump, an online vacuum degasser, an automatic liquid sampler (ALS), and a thermostated column compartment. The column employed for the separation was a Kinetex® C18 Polar (100 mm × 2.1 mm i.d.; 2.6 μm particle size) by Phenomenex (Torrance, CA, USA). Electrospray ionization (ESI) was used as an ion source, and the detection of the analytes was carried out in dynamic-multiple reaction monitoring (d-MRM). The separation was carried out with two different chromatographic methods [25]. Briefly, BPA, estrogens, and TCS were analyzed using a gradient of ultra-pure water and ACN, and the MS detection was performed in negative ionization mode. The remaining analytes were detected using a chromatographic gradient of ultra-pure water and ACN, both containing 0.001% of acetic acid and using MS polarity switching. Details on the MS parameters are reported in Table S2, along with details on the acquisition times and number of scans to guarantee a good peak shape.

## Results and discussion

### Comparison of the different materials

#### Morphology (SEM)

**Fig. 1:**
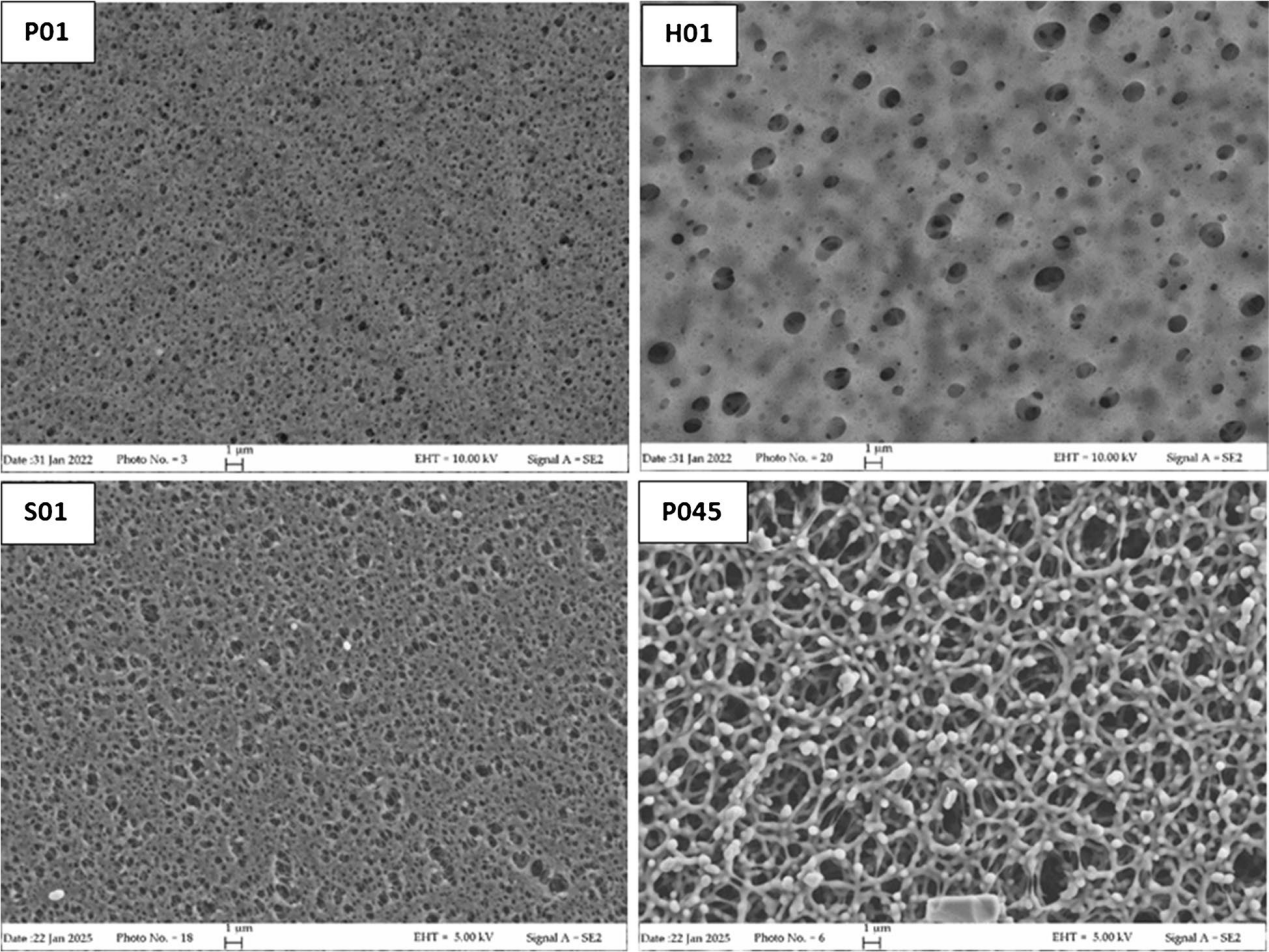
SEM images of the outer surface of the four membranes investigated: Pall Italia 0.1 μm pore size (P01), Hangzhou Anow Microfiltration 0.1 μm pore size (H01), Sterlitech Corporation 0.1 μm pore size (S01), and Pall Italia 0.45 μm pore size (P045)

**Fig. 2:**
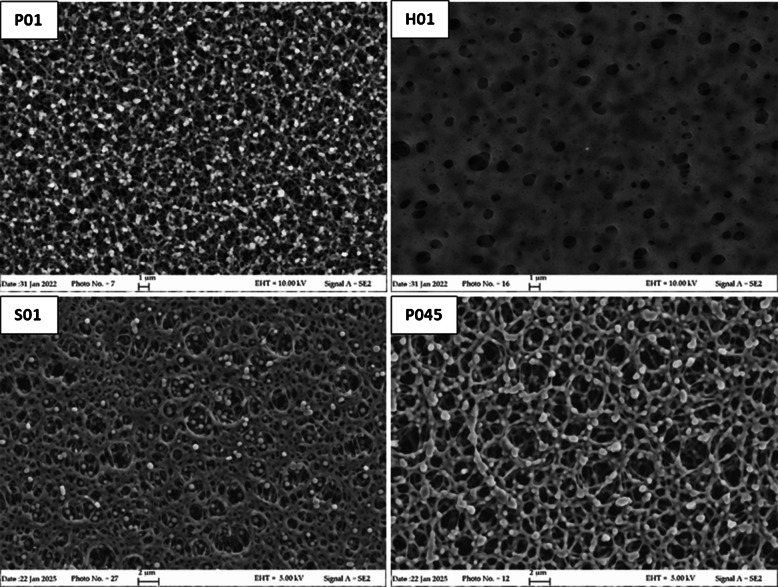
SEM images of the inner surface of the four membranes investigated: Pall Italia 0.1 μm pore size (P01), Hangzhou Anow Microfiltration 0.1 μm pore size (H01), Sterlitech Corporation 0.1 μm pore size (S01), and Pall Italia 0.45 μm pore size (P045)

**Fig. 3:**
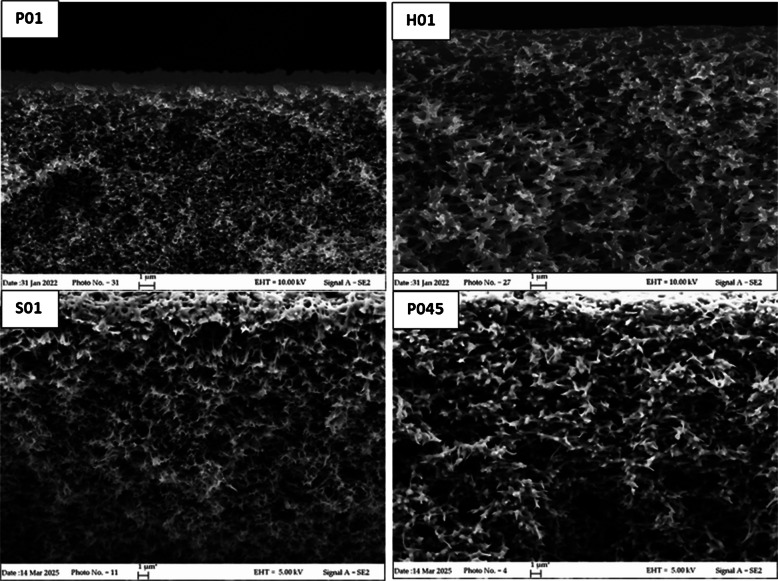
SEM images of cross-section of the four membranes investigated: Pall Italia 0.1 μm pore size (P01), Hangzhou Anow Microfiltration 0.1 μm pore size (H01), Sterlitech Corporation 0.1 μm pore size (S01), and Pall Italia 0.45 μm pore size (P045)

The four PES membrane objects of this study were analyzed by SEM to obtain information regarding their morphology. Images of the outer and inner surfaces and of the cross-sections of PES membranes are provided in Figs. 1, 2, and 3. P01 membranes have an asymmetrical structure: a smooth outer surface and a rough and more porous inner layer. The outer surface, used in POCIS as the layer exposed to water, is denser and presents a controlled size of the pores compared to the inner surface. Regarding H01 membranes, the outer layers are symmetrical and smooth with less numerous and larger pores compared to P01. Nevertheless, the nominal pore size of the membrane (the predominant pore size) could differ from the dimensions of the observed surface pores [26]. The third membrane with 0.1 µm pore size, S01, showed an asymmetrical surface with pores of controlled dimensions, like P01. Finally, the larger pore size P045 is symmetrical with clearly larger pores. The cross-section (Fig. 3) showed an even distribution of pores for all membranes, with a sponge-like morphology; however, the average dimension of the internal pores resulted in P01 < S01 < H01. This suggests a different surface area exposed to water, thus different adsorption capacity.

#### Functional groups (ATR-FTIR)

ATR-FTIR spectra were acquired to obtain information regarding the functional groups on the surface of the membrane (Fig. S1). The peaks at 1146, 1235, and 1320 cm^−1^ can be assigned to the symmetric stretching vibrations of Ar-SO_2_-Ar, the stretching of Ar-O-Ar, and the asymmetric stretching of Ar-SO_2_-Ar, respectively [27]. The peak at 1297 cm^−1^ was also assigned to the stretching of S = O [27]. These peaks, characteristic of the chemical structure of PES, showed similar intensity for all the investigated membranes. The main difference between the spectra of the PES membranes was observed for the peak at roughly 1674 cm^−1^. This signal is probably due to C = O stretching of residues of tertiary amide. In fact, N,N-dimethylacetamide and N,N-dimethylformamide are the solvents usually employed for the manufacturing of PES membranes [2]. In addition, this peak appears in the spectra of lab-modified PES membranes when N,N-dimethylacetamide is employed as solvent [28]. Furthermore, the absence of this signal in membrane with a larger pore size (P045 membranes) corroborates the hypothesis of solvent residual [29].

The information obtained from the infrared spectroscopy did not show significant variation in the surface chemistry of the membranes.

#### Porosity and hydrophilicity

To evaluate the porosity of the membranes, the thickness was initially measured using a micrometer. The thickest membrane was P045, which presented a value of 145 µm, followed by P01 and S01, both with a thickness of 135 µm. H01 had a thickness of 115 µm, resulting in the thinnest membrane. Based on the gravimetric measurements, the porosity (free volume) estimated for the analyzed membranes was 0.82 for P01, 0.80 for H01, 0.79 for S01, and 0.78 for P045. The porosity and thickness values obtained for the H01 membrane agreed with the declared information of the manufacturer (porosity of 79% and thickness of 110 μm). For P01, a lower value of porosity was reported (0.7) in a previous work [30, 31]. No information was found for the other types of membranes regarding porosity.

The hydrophilicity of PES was evaluated by the measurement of the water contact angle; a value lower than 90° was observed indicating the hydrophilic nature of all the tested membranes.

### Recovery and matrix effects

**Fig. 4:**
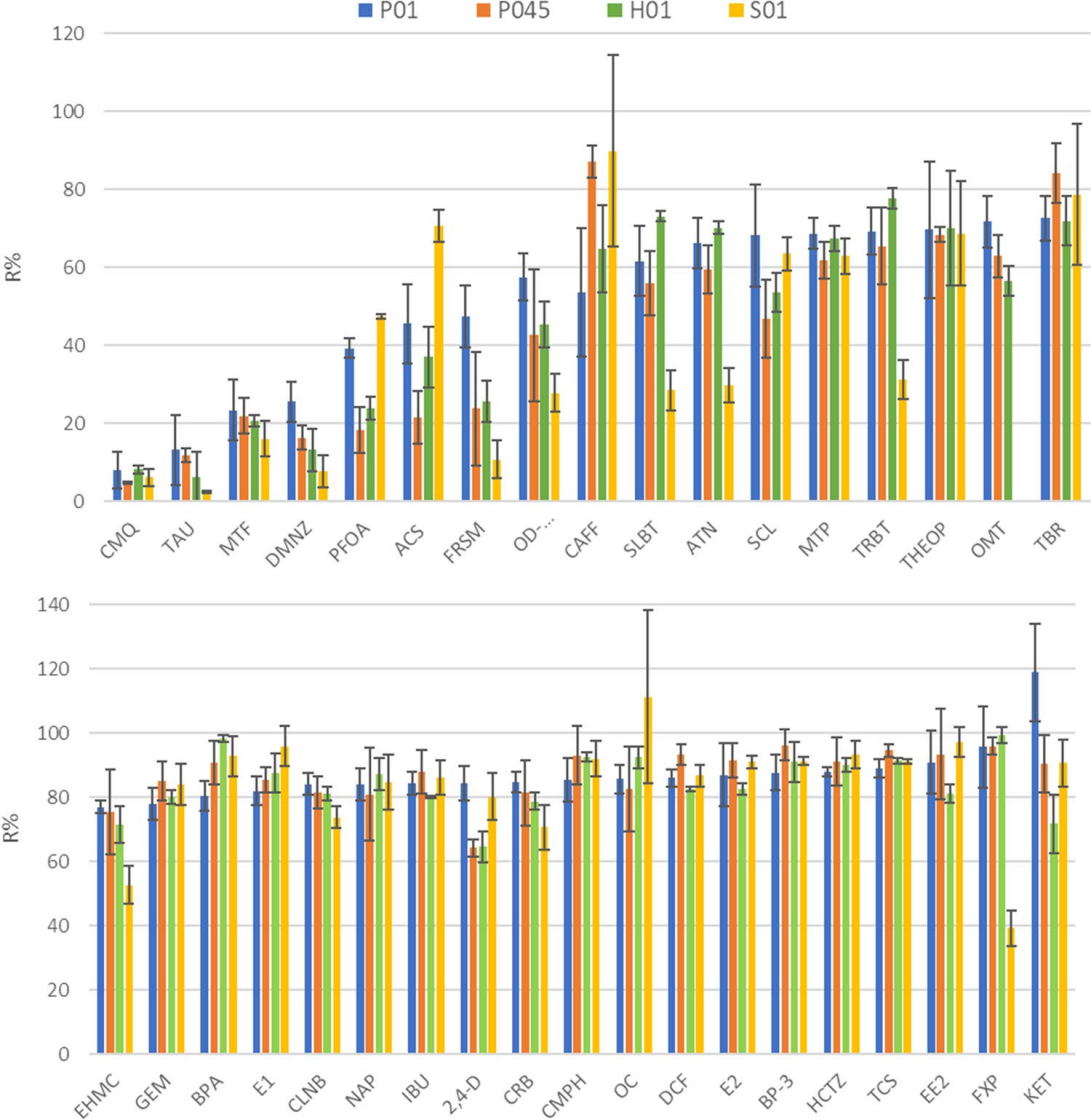
Recoveries obtained for P01 (blue), P045 (orange), H01 (green), and S01 (yellow) membranes. Error bars represent the relative standard deviation (*n* = 3)

Recoveries were mostly under 50% for ACS, TAU, DMNZ, PFOA, FRSM, MTF, CMQ, and OD-PABA (Fig. 4 and Table S3). These poor R% were mostly due to the low affinity of the target compounds for the membranes.

Considering the three membranes with the same declared pore size, the greatest differences were observed between S01 and the other types. Lower R% were obtained using S01 membranes for ATN, SLBT, TRBT, OD-PABA, EHMC, OMT, and FXP [29]. In particular, ATN, SLBT, TRBT, and FXP were detected in the rinsing water (between 33 and 63%); these compounds are charged at the working pH (Table S1), thus electrostatic repulsion could occur [2]. On the contrary, ACS, PFOA, OC, and CAFF showed the highest averaged recoveries.

On the other hand, R% of P01 and H01 mainly agreed, although a higher loss was observed in the rinsing water of H01 for PFOA, 2,4-D, and SCL. Low differences were observed between Pall membranes of different pore sizes; recoveries were higher for P01 for ACS, SCL, FRSM, PFOA, and 2,4-D, also due to higher loss in water for P045.

Alongside recovery, the evaluation of ME is crucial for the reliability of an analytical method involving HPLC-ESI-MS/MS as it can affect the precision, accuracy, and sensitivity (Taylor, 2005). This phenomenon occurs when components of the sample matrix coelute with the analytes, altering the ionization efficiency by suppression or enhancement. Thus, the presence of additives or unreacted species not completely removed by membrane pre-wash can interfere with the ionization of the compounds of interest [2].

ME% values are reported in Table S4: a soft or moderate matrix effect was observed for most compounds. Nevertheless, in the H01 and S01 membranes’ extracts, some of the more hydrophilic compounds exhibited a strong ion suppression (ME < 50%). In the case of P01 and S01, a strong ion suppression was observed for OC [29]. This result was reported also in a previous work on the use of P01 membranes as single-phase passive samplers [14].

### Partition coefficients

To assess K_PESw_ of P01—the most employed in POCIS applications—two methods were employed as reported in section “Batch experiments”: the single-dose design and the cosolvent method.

Prior to the evaluation of partition coefficients, the stability of the analytes under experimental conditions was tested (Table S5). Four target compounds showed a loss higher than 30% after 14 days (S1 setup) in water: DMNZ, HCTZ, OD-PABA, and EHMC. After 21 days of exposure using the S2 setup, also IBU, KET, and OC showed losses between 31 and 47%. Among all the analytes, only DMNZ undergoes a significant degradation for the aims of K_PESw_ evaluation. Thus, considering both its poor recovery and its poor stability, DMNZ was discarded from the list of studied compounds.

#### Single-dose design: S1 setup

**Fig. 5:**
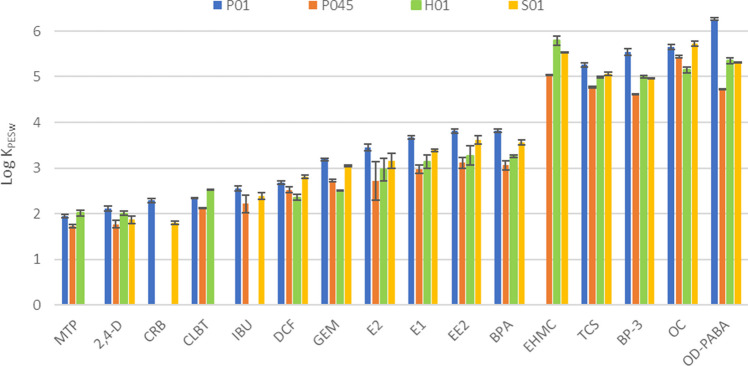
LogK_PESw_ obtained using S1 setup for the investigated PES membranes: P01, P045, H01, and S01. The error bars represent the standard deviation

Using the S1 setup, the partition coefficients were evaluated only for 16 analytes (Fig. 5), due to negligible accumulation onto the membranes for the other compounds. Two replicates were independently performed for each type of membrane (see section “Single-dose design”), mostly obtaining good repeatability (RSD% < 21%).

The sorption ability of the membrane mostly followed the order P01 > S01 > H01 > P045. Considering P01 and H01 membranes, differences in the values of LogK_PESw_ ≥ 0.5 were observed for BP-3, GEM, OD-PABA, and OC. For P01 and S01 membranes, only BP-3 and OD-PABA showed significant differences in LogK_PESw_ (0.58 and 0.96, respectively). The differences observed for OD-PABA could be attributed to the experimental variability due to its low stability under the experimental conditions.

The partition coefficient of EHMC for P01 was not estimated due to its low concentration in water after the 14-day deployment; the sorption was compared observing the amount of analyte accumulated in the membrane (Table S6). The accumulation of EHMC followed the same order presented above P01 > S01 > H01 > P045.

The different sorption ability of the membranes could be ascribed to the different distribution of pore size of H01 as observed by SEM images (Figs. 1, 2, and 3). Furthermore, H01 membranes presented the more intense peak at 1674 cm^−1^ associated with the presence of residues of tertiary amides that could hinder the access to some of the sorption sites of the membranes [29].

Regarding P045 membranes, a significant difference was mostly observed compared to the other tested membranes for the 16 analytes (> 0.5 Log units), suggesting the influence of pore size and the surface area in sorption onto PES, as highlighted in a previous study [18].

Satisfactory mass balances (≥ 70%), estimated by considering the mass extracted by the PES and the mass remained in water after the exposure, were obtained for the target compounds, excluding those that showed degradation in the control: OD-PABA and EHMC.

#### Single-dose design: S2 setup

Regarding the results obtained by the single-dose design described in section “Single-dose design: S1 setup,” too low accumulation was observed for the more polar compounds when a large volume of water (2 L) spiked at 10 µg L^−1^ was employed. Therefore, to obtain K_PESw_ for all the compounds, the S2 setup was employed: a smaller volume of water (20 mL) and a higher concentration was employed (100 µg L^−1^), as reported in section “Batch experiments.”

Considering the S1 setup, a total of 15 K_PESw_ values were obtained (the partition coefficient of EHMC using P01 membranes was not evaluated due to the low concentration in water after the exposure). Nevertheless, performing the experiments with the S2 setup, K_PESw_ values were obtained for a total of 22 compounds. Performing the experiment with the two different setups (S1 and S2) allowed obtaining a total of 29 PES-water partition coefficients. The PES-water partition coefficients were evaluated with both setups for seven compounds. Some differences were observed comparing the LogK_PESw_ obtained using the S1 and S2 setups (Table S7). In particular, GEM, IBU, DCF, and CRB showed differences of 0.42–0.66 Log units; however, considering the standard error associated with the estimated partition coefficients, it can be regarded as negligible. On the contrary, the target compounds 2,4-D, MTP, and CLBT showed a difference lower than or equal to 0.35. Excluding 2,4-D, higher values of K_PESw_ were obtained by the S2 method [29]. This may be due to the longer exposure in S2 and a possible partial non-achievement of equilibrium in S1 (apparent equilibrium due to slow accumulation). Still, considering that the K_PESw_ values obtained using the S2 setup were characterized by a higher standard deviation, the results of the S1 setup mostly fall within the S2 setup range.

#### Partition coefficients correlation with physico-chemical properties

The LogK_PESw_ obtained were then correlated with the physico-chemical properties of the target compounds. To explore the nature of the data, the PCA was employed. Twenty-nine analytes were used as objects and 13 physico-chemical properties as the variables describing the system. The first two principal components (PCs) explained 60.8% of the total variance, with PC1 accounting for 40.7% of the variance. The loading plot showed a correlation along PC1 between the variables that mainly describe hydrophobicity (e.g., polarizability, apolar surface area and the octanol-water partition coefficients at pH 5.5).

Considering the score plot, PC1 explained a gradient of LogK_PESw_ values, as shown in Fig. S2 by the color scale, with the analytes characterized by higher LogK_PESw_ showing higher PC1 scores. Thus, the main driving force of sorption seemed to be hydrophobicity and hydrophobic interaction [29].

**Fig. 6:**
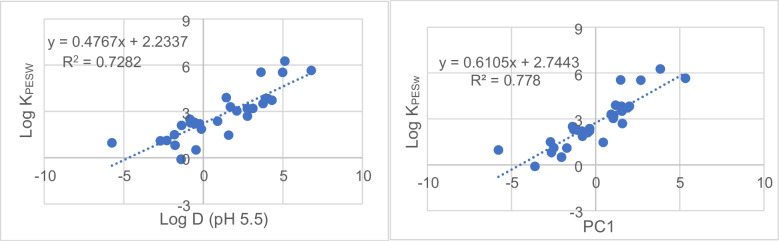
Linear correlation between the K_PESw_ obtained for the target compounds and the octanol-water partition coefficients (LogD) at pH 5.5 (left) or PC1 (right)

This observation was then investigated comparing the relationship between the LogD (at pH = 5.5) and the LogK_PESw_, obtaining an acceptable linear correlation (R_LogD_^2^ = 0.73). This correlation was then compared with the correlation between the scores of PC1 and LogK_PESw_. The correlation obtained agreed (R_PC1_^2^ = 0.78) since PC1 is mainly influenced by the variables that describe hydrophobicity, as stated before (Fig. 6). Nonetheless, considering a reduced range of polarities, some exceptions related to the chemical structure can be highlighted, as previously stated [14].

#### Cosolvent method

The cosolvent method was employed for the first time in this work to evaluate K_PESw_. This design is usually employed for hydrophobic compounds to evaluate polymer-water partition coefficients in SR. Different percentages of an organic solvent are added to the aqueous phase in order to increase the compound solubility in the solvent. Consequently, the higher the organic fraction present in the mixture, the lower the partition coefficients obtained. Still, the higher equilibrium concentrations in water permit a more accurate quantification.

Acceptable average mass balances were observed for the target compounds using the cosolvent experiment (77–121%); the only exception was KET, with an average mass balance of 64%, which can be explained by the 40% loss observed in the blank experiment (Table S5). The four investigated UV filters and TCS were not considered because of their exhaustive sorption onto PES, due to the small volume employed and their high sorption affinity for the polymer [29].

**Fig. 7:**
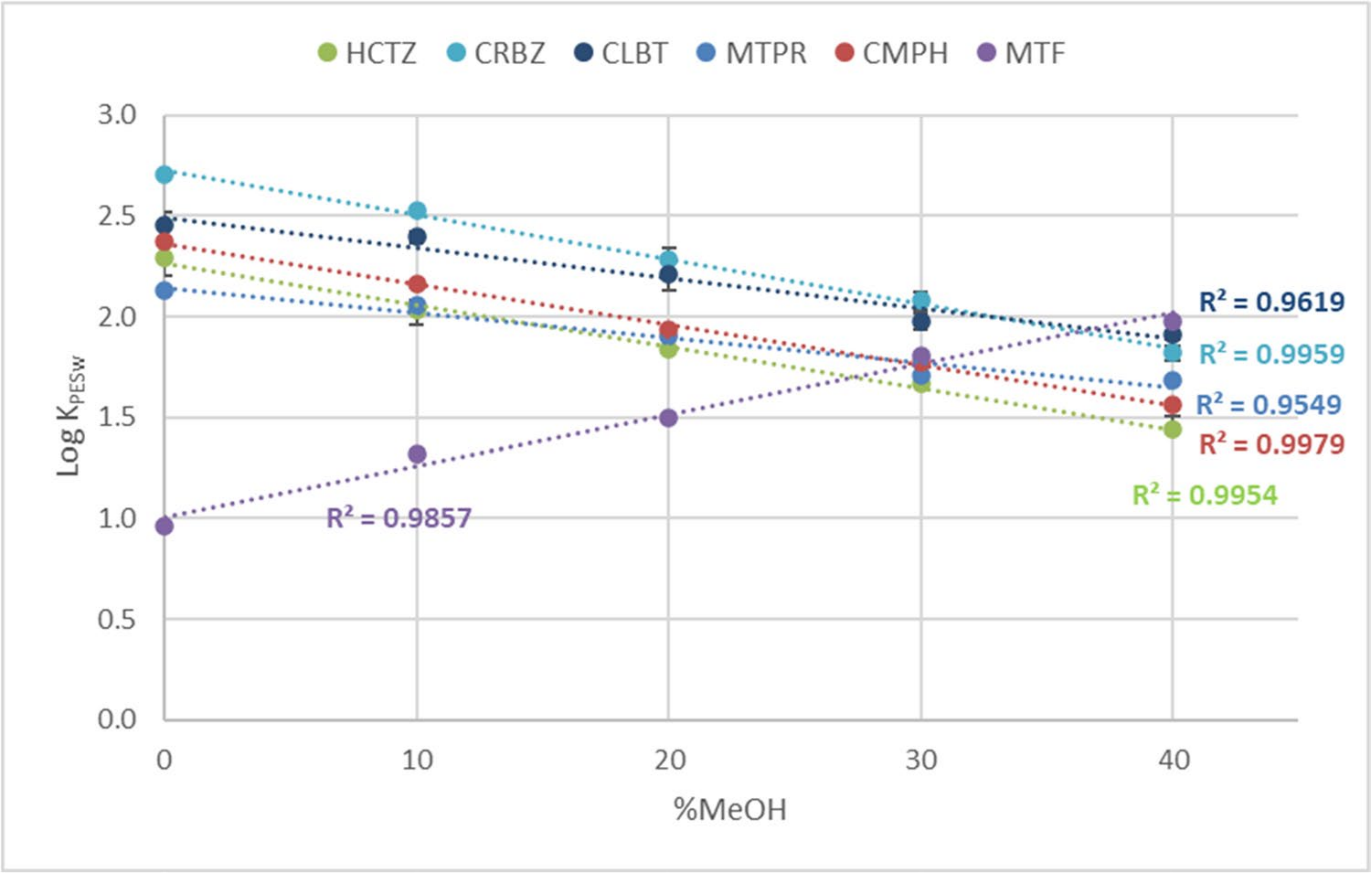
Examples of linear regressions obtained for the target compounds showing a linear increase or decrease of LogK_PESw_ as a function of the %MeOH added as cosolvent

Usually, the cosolvent is employed to estimate partition coefficients for the more hydrophobic compounds to avoid their sorption on the walls of the container and on the dissolved organic matter. However, the analytes investigated here covered a wide range of Log K_ow_. The K_PESw_ obtained at different % MeOH in the mixture were plotted and linear correlation was observed for 11 compounds (Fig. 7). The analytes showing this linear profile were E2, EE2, E1, BPA, THEOP, PFOA, HCTZ, CRB, CLBT, MTF, CHMP, and MTP, which presented regression coefficients ranging from 0.817 to 0.998. Nevertheless, MTF presented the opposite behavior: the K_PESw_ values increased by increasing the % of MeOH. This compound is the most polar of the target group (LogD =−5.59), thus lowering the polarity of water by adding crescent molar fractions of MeOH may have promoted the sorption onto the PES membrane.

Another group of analytes did not show significant differences as a function of the MeOH molar fraction in the mixture. This smaller group includes ATL, SLBT, and TRBT, which presented changes in LogK_PESw_ by varying the % of MeOH in the mixture, lower than 0.2 Log unit (also considering experimental variability). Finally, the group containing 2,4-D, FRSM, DCF, KET, IBU, GEM, and NAP did not present a linear decrease in K_PESw_ when increasing the % MeOH. However, compared to ATL, SLBT, and TRBT, higher differences were observed between the K_PESw_. Furthermore, these compounds presented a decrease in the value of the partition coefficient corresponding to 10% of MeOH.

The LogK_PESw_ evaluated for the compounds presenting a linear trend using the cosolvent method are reported in Table S7. The linear regression was performed using the values obtained with 10–40% of methanol and then comparing the intercept (extrapolated LogK_PESw_) to the zero cosolvent values (corresponding to the S2 setup). The results agreed with the LogK_PESw_ reported using the S2 setup. Regarding the partition coefficient obtained using the S1 setup, higher differences can be observed. In particular, BPA, E1, and E2 presented a gap between 0.34–0.8 Log unit. These could be due to changes in the slope of LogK_PESw_ vs. % methanol at low concentration of methanol [23].

#### Comparison with literature data

Three previous works have attempted to develop poly-parameter linear free energy relationships (pp-LFER) for K_PESw_ fitting or prediction [5, 13, 16]. In these models, Abraham’s descriptors were employed: excess molar refractivity (describing part of Van der Waals force), polarizability, hydrogen bond acidity/donating capacity, hydrogen bond basicity/accepting capacity and McGowan molar volume (molecule’s size). The first two models developed presented poor fit [13, 16], while the latest one presented a good description of the data [5]. Improvements in fitting were obtained using a larger dataset (125 compounds vs. 21 and 90), but moderate predictability was shown and for this reason the model was suggested only as a screening tool.

One of the reasons for the unsatisfactory results can be ascribed to the weak accuracy of some of the K_PESw_ employed. In fact, several produced partition coefficients were only apparent because the achievement of the equilibrium was not verified, or in the worst case the equilibrium was not reached.

**Fig. 8:**
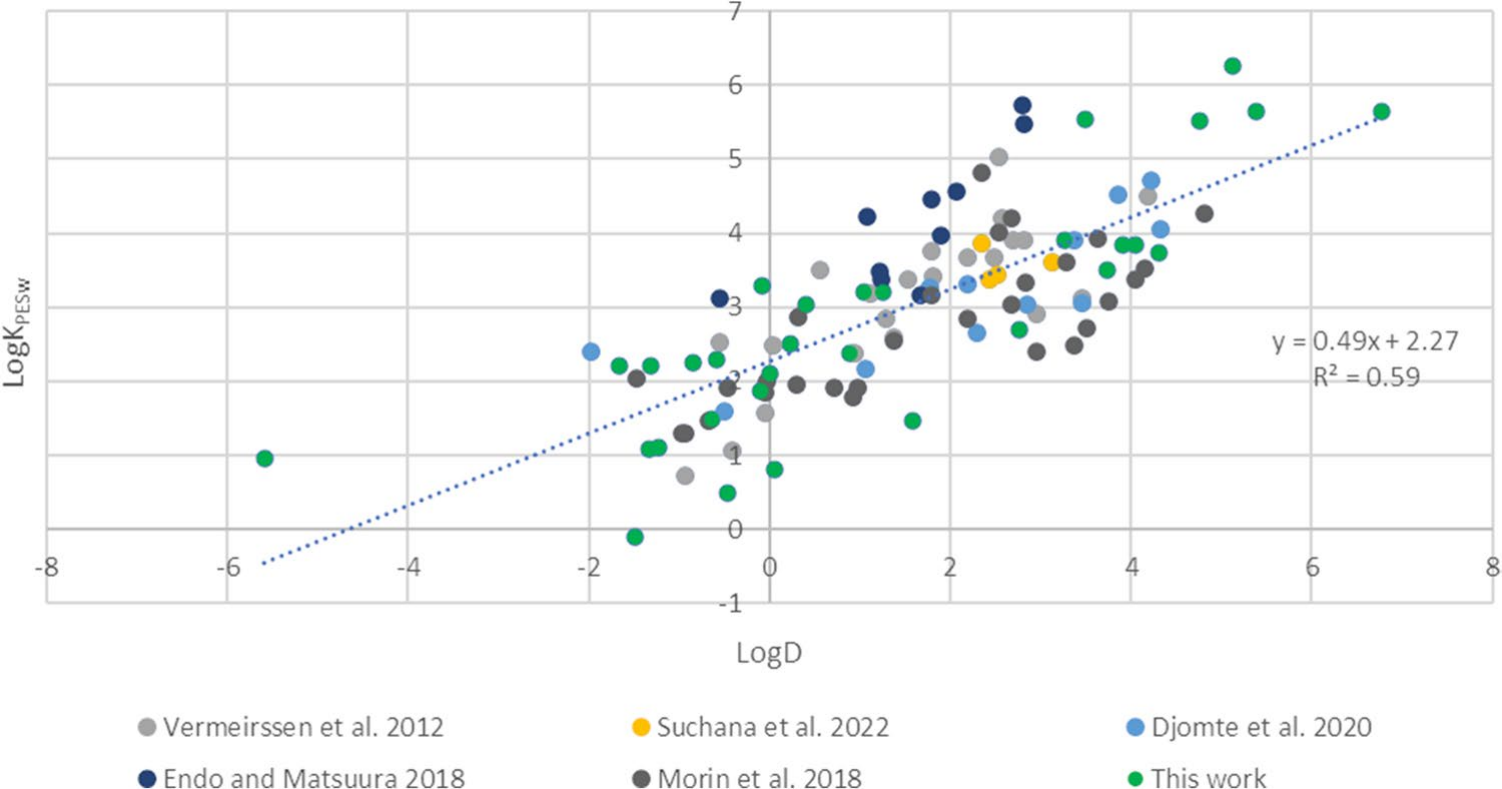
Linear regression obtained plotting 77 LogK_PESw_ presented in literature and the P01 value obtained in this work (green dots) against the LogD of the compound (pH 7 or pH 5.5 depending on the experimental conditions). A total of 91 compounds were considered

Based on the results presented in Fig. 6, the K_PESw_ herein obtained result correlated to the distribution coefficient (LogD) of the target chemicals. Seventy-seven K_PESw_ presented in the literature and obtained using PES sheets of 0.1 µm pore size both in single-phase [5, 12, 15] and dual-phase [11] configurations were considered together with the data presented in this work. The aim was to observe the correlations between K_PESw_ values and the LogD of chemicals. The resulting linear regression (Fig. 8) showed a moderate correlation (*R*^2^ = 0.59) and a similar relationship to the one reported in Fig. 6.

**Fig. 9:**
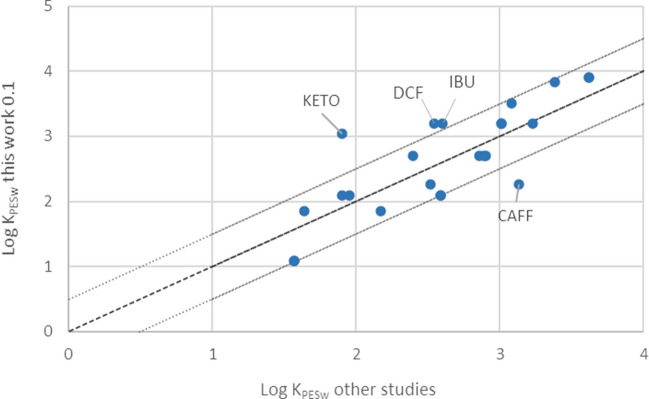
Graph of the LogK_PESw_ reported in literature for PES membranes of 0.1 µm (*x*-axis) and those obtained in this work for P01 membranes (*y*-axis). The dotted lines mark the range where compared values differ by less than a 0.5 factor

In addition, the K_PESw_ obtained herein were compared to those reported in previous studies for different PES membranes even when the equilibrium was not verified [4, 9–11, 15]. In Fig. 9, the results of this work were plotted against K_PESw_ found in literature for PES membranes of 0.1 µm. The K_PESw_ were mainly included between the dotted line representing a difference of 0.5 Log units. Moreover, the K_PESw_ values present in literature for PES membranes of larger pore size (0.45 µm and 0.2 µm) [10] and those obtained in this work using P045 were compared for HCTZ, 2,4-D, and TCS (the sole common analytes); only TCS showed differences lower than 0.5 Log units while the gap for HCTZ and 2,4-D was higher. Still, this difference may be ascribed to the mode used in this single study to evaluate the partition coefficient [29].

Prieto et al. (2012) reported K_PESw_ for several compounds using PES tubes for sorptive microextraction [32]. These partition coefficients were estimated in the presence of 20% of MeOH and expressed in [L L^−1^]. The K_PESw_ of the analytes common to both studies (E2, CAFF, TCS, OD-PABA and OC) were compared. In particular, the partition coefficients of CAFF and E2 were comparable considering the values obtained using the cosolvent method in the presence of 20% MeOH. The results differ by 0.1 Log units for CAFF and 0.53 for E2. On the other hand, the results of OD-PABA, OC, and TCS could be compared only with those obtained using the S1 setup, in the absence of MeOH. As expected, the values reported for the present work were higher (by 1.4 Log units). As mentioned before, in the presence of an organic solvent the partition coefficients between the polymer and the mixture are lower than in the presence of the sole water, for hydrophobic compounds [29].

## Conclusions

The affinity for PES membranes of 36 target compounds characterized by a broad range of physico-chemical properties was investigated. The PES-water partition coefficients of the analytes were assessed for 29 compounds using two different methods: the single-dose design and the cosolvent method. Both methodologies presented advantages and could be useful to limit some of the problems related to the evaluation of partition coefficients. The impact of PES manufacturing on analyte’s sorption was also studied for 16 analytes using membranes of the same declared pore size (0.1 µm), purchased from three different producers. The results obtained showed significant differences in sorption, underlining the importance of evaluating the affinity of the specific PES protective membrane in dual-phase passive samplers, to avoid inaccuracy in the estimation of pollutant’s water concentrations. The influence of pore size on the accumulation was also analyzed comparing K_PESw_ of membranes with pores of 0.1 and 0.45 µm, showing a greater sorption capacity of PES with lower pore dimension.

Considering the results obtained for the most employed PES membrane in passive sampling (P01), a correlation between the K_PESw_ and the physico-chemical properties of the analytes was evaluated using the principal component analysis. The results indicated hydrophobicity as the main driving force of compounds’ affinity for PES. The comparison of the present results with the literature highlighted the necessity of a more uniform evaluation strategy, in order to obtain comparable and reliable K_PESw_. This would allow a more informed choice of the target analytes when using PES in both dual and single-phase passive samplers. Also, the use of PES membranes as a protective layer can be useful to widen the number of compounds detectable using dual-phase passive samplers, exploiting both the sorption by the sorbent and the membrane. In this scenario, a separate extraction of the two compartments would permit the evaluation of the environmental concentration of those analytes with high affinity for PES and negligible sorption by the sorbent.

## Supplementary Information

Below is the link to the electronic supplementary material.

Supplementary file1 (DOCX 393 KB)

## Data Availability

The data of this study are included in the article. Additional data will be made available upon request.
