# Supplementary material for: Sorption properties of several polyethersulfone membranes for hydrophilic and hydrophobic emerging contaminants: implications for their application in passive sampling
Source: Anal Bioanal Chem. 2026 Apr 20;418(12):3723–36. doi: 10.1007/s00216-026-06476-4 (PMC13221335; doi:10.1007/s00216-026-06476-4)
Supplement: Supplementary file 1 — Supplementary file1 (DOCX 393 KB) [file 216_2026_6476_MOESM1_ESM.docx]

**Supporting Information**

Sorption properties of several polyethersulfone membranes for hydrophilic and hydrophobic emerging contaminants: implications for their application in passive sampling

Chiara Scapuzzi, Henry MacKeown, Barbara Benedetti, Marina Di Carro, Emanuele Magi*.

University of Genoa, Department of Chemistry and Industrial Chemistry, Via Dodecaneso 31, 16146 Genoa, Italy

* corresponding author: [emanuele.magi@unige.it](mailto:emanuele.magi@unige.it)

**Tab. S1** Physico-chemical properties of the target compound of this studys: LogD at pH 5.5 (LogD5.5), Molecular Weight (MW), Number of H-bonding donor groups (Hbd), Number of H-bonding acceptor groups (Hba), Topological Polar Surface Area (TPSA), Number of Aromatic Rings (AR), Polarizability (Pol), Solvent Accessible Surface Area (SASA), Apolar Surface Area (Apolar SA), Solubility (LogS), percentage of neutral analyte at pH = 5.5 (N%), percentage of anionic analyte at pH = 5.5 (A%), percentage of cationic analyte at pH = 5.5 (C%).

| **Analyte** | **LogD7.5** | **LogD5.5** | **MW** | **HBd** | **Hba** | **TPSA** | **AR** | **Pol** | **SASA** | **LogS** | **Apolar SA** | **N%** | **A%** | **C%** |
| --- | --- | --- | --- | --- | --- | --- | --- | --- | --- | --- | --- | --- | --- | --- |
| **MTF** | -5.59 | -5.75 | 129.2 | 4 | 5 | 88.99 | 0 | 13.0 | 278.3 | 1.8 | 189.4 | 0 | 0 | 100.00 |
| **CMQ** | -3.31 | -3.31 | 122.6 | 0 | 0 | nd | 0 | 13.2 | 286.9 | nd | nd | 0 | 0 | 100.00 |
| **ATN** | -1.33 | -2.70 | 266.3 | 3 | 4 | 84.58 | 1 | 29.1 | 535.7 | 0.2 | 451.2 | 0 | 0.02 | 99.98 |
| **TAU** | -2.62 | -2.62 | 125.1 | 2 | 4 | 80.39 | 0 | 10.7 | 282.7 | 1.0 | 202.3 | 99.99 | 0.01 | 0 |
| **DMNZ** | -4.23 | -2.38 | 160.2 | 2 | 4 | 69.64 | 0 | 15.0 | 373.8 | 2.6 | 304.2 | 9.07 | 90.92 | 0 |
| **SLBT** | -1.23 | -2.31 | 239.3 | 4 | 4 | 72.72 | 1 | 26.6 | 463.9 | 1.2 | 391.2 | 0.01 | 0 | 99.99 |
| **TRBT** | -0.64 | -1.82 | 225.3 | 4 | 4 | 72.72 | 1 | 24.7 | 436.6 | 1.1 | 363.9 | 0.04 | 0 | 99.96 |
| **PRX** | -1.32 | -1.61 | 180.2 | 1 | 5 | 68.50 | 2 | 16.5 | 295.31 | -0.2 | 226.8 | 28.23 | 0 | 71.56 |
| **ACS** | -1.49 | -1.40 | 163.2 | 1 | 4 | 72.47 | 0 | 13.5 | 304.69 | 0.7 | 232.2 | 3.18 | 0 | 96.82 |
| **MTP** | -0.001 | -1.37 | 267.4 | 2 | 4 | 50.72 | 1 | 30.3 | 602.12 | 0.3 | 551.4 | 0 | 0.02 | 99.98 |
| **CLBT** | 0.23 | -0.85 | 277.2 | 3 | 3 | 58.28 | 1 | 28.4 | 448.13 | 0 | 389.9 | 0.01 | 0 | 99.99 |
| **THEOP** | -0.85 | -0.81 | 180.2 | 1 | 3 | 69.30 | 2 | 16.1 | 316.7 | -1.0 | 247.4 | 99.80 | 0 | 0.20 |
| **FXP** | -1.87 | -0.68 | 255.0 | 2 | 5 | 85.44 | 1 | 19.4 | 382.9 | 0 | 297.5 | 0.51 | 99.49 | 0 |
| **HCTZ** | -0.59 | -0.58 | 297.7 | 3 | 5 | 118.36 | 1 | 25.3 | 419.9 | -1.8 | 301.6 | 99.97 | 0.03 | 0 |
| **OMT** | -0.55 | -0.55 | 213.2 | 1 | 2 | 64.63 | 0 | 19.1 | 446.8 | -0.7 | 382.2 | 100.00 | 0 | 0 |
| **CAFF** | -0.55 | -0.55 | 194.2 | 0 | 3 | 58.44 | 2 | 17.9 | 368.4 | -0.4 | 309.9 | 100.00 | 0 | 0 |
| **SCL** | -0.47 | -0.47 | 397.6 | 5 | 8 | 128.84 | 0 | 32.7 | 422.5 | -2.3 | 293.6 | 100.00 | 0 | 0 |
| **FRSM** | -1.66 | -0.24 | 330.7 | 3 | 5 | 122.63 | 2 | 29.5 | 503.9 | 0 | 381.3 | 1.00 | 99.00 | 0 |
| **2,4-D** | -0.10 | -0.13 | 221.0 | 1 | 3 | 46.53 | 1 | 19.1 | 370.6 | -0.4 | 324.1 | 0.20 | 99.8 | 0 |
| **CMPH** | 0.88 | 0.88 | 323.1 | 3 | 5 | 112.7 | 1 | 27.8 | 460.5 | -3.2 | 347.8 | 100.00 | 0 | 0 |
| **GEM** | 3.27 | 1.44 | 250.3 | 1 | 3 | 46.53 | 1 | 27.9 | 533.4 | -2.2 | 486.9 | 7.63 | 92.37 | 0 |
| **PFOA** | 1.58 | 1.59 | 414.1 | 1 | 2 | 37.30 | 0 | 16.7 | 440.0 | -1.0 | 402.7 | 0 | 100.00 | 0 |
| **NAP** | -0.09 | 1.70 | 230.3 | 1 | 3 | 46.53 | 2 | 26.4 | 452.5 | -2.2 | 406.0 | 5.20 | 94.80 | 0 |
| **KET** | 0.40 | 2.10 | 254.3 | 1 | 3 | 54.37 | 2 | 28.0 | 452.3 | -2.3 | 397.9 | 3.05 | 96.95 | 0 |
| **DCF** | 1.05 | 2.76 | 296.2 | 2 | 3 | 49.33 | 2 | 29.0 | 425.0 | -2.8 | 375.6 | 0.10 | 96.89 | 0 |
| **CRB** | 2.77 | 2.77 | 236.3 | 1 | 1 | 46.33 | 2 | 27.0 | 370.9 | -3.8 | 324.6 | 100.00 | 0.00 | 0 |
| **IBU** | 1.25 | 3.11 | 206.3 | 1 | 2 | 37.30 | 1 | 23.7 | 430.1 | -2.8 | 392.8 | 18.36 | 81.64 | 0 |
| **BP-3** | 3.50 | 3.62 | 228.2 | 1 | 3 | 46.53 | 2 | 25.1 | 443.7 | -3.4 | 397.2 | 78.92 | 21.08 | 0 |
| **E2** | 3.74 | 3.75 | 272.4 | 2 | 2 | 40.46 | 1 | 31.3 | 395.1 | -4.0 | 354.6 | 99.85 | 0.15 | 0 |
| **EE2** | 3.90 | 3.90 | 296.4 | 2 | 2 | 40.46 | 1 | 33.9 | 411.2 | -4.8 | 370.8 | 99.85 | 0.15 | 0 |
| **BPA** | 4.04 | 4.05 | 228.3 | 2 | 2 | 40.46 | 2 | 26.6 | 413.1 | -3.2 | 372.6 | 99.48 | 0.52 | 0 |
| **E1** | 4.31 | 4.31 | 270.4 | 1 | 2 | 37.30 | 1 | 30.8 | 396.1 | -4.2 | 358.8 | 99.85 | 0.15 | 0 |
| **TCS** | 4.76 | 4.98 | 289.5 | 1 | 1 | 29.46 | 2 | 27.0 | 413.4 | -5.3 | 384.0 | 60.25 | 39.75 | 0 |
| **OD-PABA** | 5.12 | 5.12 | 277.4 | 0 | 2 | 29.54 | 1 | 32.5 | 623.2 | -4.8 | 593.7 | 100.00 | 0 | 0 |
| **EHMC** | 5.38 | 5.38 | 290.4 | 0 | 2 | 35.53 | 1 | 33.6 | 639.0 | -5.6 | 603.4 | 100.00 | 0 | 0 |
| **OC** | 6.78 | 6.78 | 361.5 | 0 | 2 | 50.09 | 2 | 42.4 | 646.7 | -7.2 | 596.6 | 100.00 | 0 | 0 |

**Tab. S2** Optimized MS parameters for the transitions of the target chemicals used in the dynamic MRM method.

| **Analyte** | **Formula** | **Polarity of ESI** | **RT (min)** | **Quantifier transition**  **(m/z)** | | **CE (V)** | **frag (V)** | **Qualifier transition (m/z)** | | **CE (V)** |
| --- | --- | --- | --- | --- | --- | --- | --- | --- | --- | --- |
| **ACS** | C_4_H_5_NO_4_S | ESI - | 0.64 | 162 | 82 | 12 | 94 | 162 | 78 | 36 |
| **TAU** | C_2_H_7_NO_3_S | ESI - | 0.84 | 124 | 79.9 | 24 | 96 | nd | / | / |
| **OMT** | C_5_H_12_NO_4_PS | ESI + | 0.85-1 | 214 | 155 | 12 | 68 | 214 | 125 | 20 |
| **DMNZ** | C_6_H_12_N_2_O_3_ | ESI + | 0.87 | 161.1 | 143.1 | 8 | 68 | 161.1 | 61.2 | 8 |
| **PRX** | C_7_H_8_N_4_O_2_ | ESI + | 1 | 181 | 124 | 20 | 77 | nd | / | / |
| **THEOP** | C_7_H_8_N_4_O_2_ | ESI + | 1 | 181 | 124 | 20 | 77 | nd | / | / |
| **CAFF** | C_8_H_10_N_4_O_2_ | ESI + | 1 | 195 | 138 | 20 | 100 | 195 | 110 | 20 |
| **PFOA** | C_8_HF_15_O_2_ | ESI - | 1 | 413 | 369 | 5 | 40 | 413 | 169 | 20 |
| **SCL** | C_12_H_19_Cl_3_O_8_ | ESI - | 1 | 455 | 395 | 4 | 104 | 457 | 397 | 4 |
| **HCTZ** | C_7_H_8_ClN_3_O_4_S_2_ | ESI - | 1.05 | 296 | 269 | 16 | 170 | 296 | 205 | 20 |
| **2,4-D** | C_8_H_6_Cl_2_O_3_ | ESI - | 1.2 | 219 | 160.8 | 8 | 68 | 219 | 124.9 | 28 |
| **FRSM** | C_12_H_11_ClN_2_O_5_S | ESI - | 1.3 | 329 | 285 | 12 | 90 | 329 | 205 | 20 |
| **CMPH** | C_11_H_12_Cl_2_N_2_O_5_ | ESI - | 1.34 | 321 | 257 | 4 | 96 | 321 | 151.9 | 12 |
| **CRB** | C_15_H_12_N_2_O | ESI + | 1.76 | 237 | 194 | 20 | 120 | 237 | 165 | 48 |
| **KET** | C_16_H_14_O_3_ | ESI - | 2.7-2.9 | 253 | 209 | 2 | 40 | nd | / | / |
| **NAP** | C_14_H_14_O_3_ | ESI - | 2.8-3 | 229 | 170 | 5 | 40 | 229 | 169 | 15 |
| **MTF** | C_4_H_11_N_5_ | ESI + | 3.3-3.8 | 130.1 | 68.1 | 40 | 96 | 130.1 | 60.1 | 12 |
| **ATN** | C_14_H_22_N_2_O_3_ | ESI + | 3.4-4 | 267.2 | 145.1 | 28 | 152 | 267.2 | 56.2 | 36 |
| **SLBT** | C_13_H_21_NO_3_ | ESI + | 3.6-4.2 | 240 | 148 | 16 | 96 | 240 | 222 | 8 |
| **TRBT** | C_12_H_19_NO_3_ | ESI + | 3.7-4.4 | 226.1 | 152.1 | 16 | 96 | 226.1 | 107.1 | 32 |
| **CMQ** | C_5_H_13_ClN | ESI + | 3.7-4.4 | 122.1 | 59.2 | 20 | 96 | 122.1 | 58.2 | 36 |
| **DCF** | C_14_H_11_Cl_2_NO_2_ | ESI - | 4.8 | 294 | 250 | 5 | 40 | 294 | 214 | 20 |
| **IBU** | C_13_H_18_O_2_ | ESI - | 5 | 205 | 161 | 2 | 40 | nd | / | / |
| **BP-3** | C_14_H_12_O_3_ | ESI + | 5.4 | 229 | 151 | 16 | 116 | 229 | 105 | 16 |
| **GMF** | C_15_H_22_O_3_ | ESI - | 5.6-5.7 | 249 | 121 | 4 | 110 | 249 | 106 | 50 |
| **FXP** | C_7_H_5_Cl_2_FN_2_O_3_ | ESI + | 6.6-7.6 | 255 | 209 | 16 | 96 | 255 | 181 | 24 |
| **MTP** | C_15_H_25_NO_3_ | ESI + | 7.4-8.4 | 268.2 | 74.1 | 24 | 124 | 268.2 | 56.2 | 32 |
| **CLBT** | C_12_H_18_Cl_2_N_2_O | ESI + | 8.3-10.1 | 277 | 203 | 12 | 80 | 277 | 132 | 32 |
| **OD-PABA** | C_17_H_27_NO_2_ | ESI + | 8.9 | 278 | 151 | 32 | 116 | 278 | 166 | 20 |
| **EHMC** | C_18_H_26_O_3_ | ESI + | 9.06 | 291 | 161 | 16 | 60 | 261 | 179 | 4 |
| **EHS** | C_15_H_22_O_3_ | ESI + | 9.33 | 251 | 139 | 4 | 76 | nd | / | / |
| **OC** | C_24_H_27_NO_2_ | ESI + | 9.6-9.7 | 362 | 250 | 8 | 152 | 362 | 232 | 20 |
| **BPA** | C_15_H_16_O_2_ | ESI - | 2.6 | 227 | 212 | 12 | 128 | 227 | 133 | 24 |
| **E2** | C_18_H_24_O_2_ | ESI - | 3 | 271 | 145 | 40 | 180 | 271 | 183 | 40 |
| **EE2** | C_20_H_24_O_2_ | ESI - | 3.8 | 295 | 145 | 30 | 180 | 295 | 159 | 35 |
| **E1** | C_18_H_22_O_2_ | ESI - | 4.1 | 269 | 145 | 30 | 110 | 269 | 143 | 60 |
| **TCS** | C_12_H_7_Cl_3_O_2_ | ESI - | 6.8 | 287 | 35 | 4 | 72 | 289 | 35 | 4 |

By using Dynamic MRM, the acquisition is limited to specific MRM transitions in specific time intervals (centred on the RT of each analytes). Based on RT, peak width and maximum 2 considered MRM per chemical (each with a dwell time of 200 ms) a rather high number of scans per peak were obtained (40 on average), allowing a very good and reproducible peak shape.

**Tab. S3** Recoveries (R%) of the extraction procedure and loss in the rinsing water (W%) for all the analysed compounds using the four different PES membranes.

|  | **P01** | | **P045** | | **H01** | | **S01** | |
| --- | --- | --- | --- | --- | --- | --- | --- | --- |
|  | *R%* | *W%* | *R%* | *W%* | *R%* | *W%* | *R%* | *W%* |
| **ACS** | 46 ± 10 | 36 ± 8 | 22 ± 7 | 69 ± 9 | 37 ± 8 | 58 ± 14 | 71 ± 4 | 19 ± 2 |
| **TAU** | 13 ± 9 | 51 ± 9 | 12 ± 2 | 69 ± 19 | 6 ± 7 | 67 ± 15 | 2 ± 0 | 83 ± 7 |
| **OMT** | 72 ± 7 | 21 ± 4 | 63 ± 5 | 32 ± 1 | 57 ± 4 | 42 ± 8 | nd* | 28 ± 1 |
| **TBR** | 73 ± 6 | 8 ± 3 | 84 ± 8 | 18 ± 9 | 72 ± 6 | 18 ± 7 | 79 ± 18 | 11 ± 2 |
| **THEOP** | 70 ± 17 | 9 ± 2 | 68 ± 2 | 17 ± 7 | 70 ± 15 | 21 ± 5 | 69 ± 13 | 12 ± 1 |
| **DMNZ**** | 26 ± 5 | 40 ± 8 | 16 ± 3 | 59 ± 14 | 13 ± 5 | 57 ± 13 | 8 ± 4 | 65 ± 11 |
| **SCL** | 68 ± 13 | 20 ± 5 | 47 ± 10 | 45 ± 7 | 54 ± 5 | 43 ± 10 | 63 ± 4 | 32 ± 2 |
| **CAFF** | 54 ± 16 | 7 ± 1 | 87 ± 4 | 9 ± 4 | 65 ± 11 | 11 ± 2 | 90 ± 25 | 5.7 ± 0.4 |
| **PFOA** | 39 ± 2 | < 5% | 18 ± 6 | 9.3 ± 0.3 | 24 ± 3 | 10 ± 3 | 48 ± 1 | < 5% |
| **HCTZ** | 88 ± 1 | < 5% | 91 ± 7 | < 5% | 90 ± 2 | < 5% | 93 ± 4 | < 5% |
| **2,4-D** | 84 ± 5 | < 5% | 64 ± 3 | 25 ± 2 | 65 ± 5 | 22 ± 8 | 80 ± 7 | 7 ± 2 |
| **FRSM** | 47 ± 8 | nd | 24 ± 15 | < 5% | 26 ± 5 | < 5% | 11 ± 5 | < 5% |
| **CMPH** | 85 ± 7 | nd | 93 ± 9 | < 5% | 93 ± 2 | < 5% | 92 ± 6 | < 5% |
| **CRB** | 85 ± 3 | < 5% | 81 ± 10 | < 5% | 79 ± 3 | < 5% | 71 ± 7 | < 5% |
| **KET** | 119 ± 15 | nd | 90 ± 9 | nd | 72 ± 9 | nd | 91 ± 7 | nd |
| **NAP** | 84 ± 5 | nd | 81 ± 15 | < 5% | 87 ± 5 | < 5% | 85 ± 9 | < 5% |
| **MTF** | 23 ± 8 | 34 ± 7 | 22 ± 5 | 33 ± 4 | 21 ± 1 | 32 ± 4 | 16 ± 5 | 72 ± 11 |
| **ATN** | 66 ± 7 | 17 ± 5 | 60 ± 6 | 19 ± 2 | 70 ± 2 | 13 ± 2 | 30 ± 4 | 58 ± 9 |
| **SLBT** | 62 ± 9 | 22 ± 6 | 56 ± 8 | 24 ± 3 | 73 ± 1 | 15 ± 3 | 28 ± 5 | 57 ± 7 |
| **TRBT** | 69 ± 6 | 10 ± 3 | 65 ± 10 | 10 ± 2 | 78 ± 3 | 6 ± 1 | 31 ± 5 | 40 ± 7 |
| **CMQ** | 8 ± 5 | 49 ± 11 | 5 ± 0 | 52 ± 4 | 8 ± 1 | 44 ± 8 | 6 ± 2 | 77 ± 11 |
| **DIC** | 86 ± 3 | nd | 93 ± 3 | nd | 83 ± 1 | nd | 87 ± 4 | nd |
| **IBU** | 84 ± 4 | nd | 88 ± 7 | < 5% | 80 ± 0 | < 5% | 86 ± 5 | < 5% |
| **BP-3** | 88 ± 6 | nd | 96 ± 5 | nd | 91 ± 6 | nd | 91 ± 1 | nd |
| **GEM** | 78 ± 5 | < 5% | 85 ± 6 | < 5% | 80 ± 2 | < 5% | 84 ± 7 | < 5% |
| **FXP** | 96 ± 13 | 24 ± 5 | 96 ± 3 | 28 ± 3 | 99 ± 3 | 16 ± 3 | 39 ± 5 | 59 ± 11 |
| **MTP** | 69 ± 4 | < 5% | 62 ± 5 | < 5% | 67 ± 3 | < 5% | 63 ± 5 | 16 ± 3 |
| **CLNB** | 84 ± 3 | < 5% | 82 ± 5 | < 5% | 81 ± 2 | < 5% | 74 ± 3 | 13 ± 3 |
| **OD-PABA** | 57 ± 6 | nd | 43 ± 17 | nd | 45 ± 6 | nd | 28 ± 5 | nd |
| **EHMC** | 77 ± 2 | nd | 75 ± 13 | nd | 71 ± 6 | nd | 53 ± 6 | nd |
| **OC** | 86 ± 5 | nd | 82 ± 13 | nd | 92 ± 4 | nd | 111 ± 27 | nd |
| **BPA** | 80 ± 5 | nd | 91 ± 7 | nd | 98 ± 1 | nd | 93 ± 6 | nd |
| **E2** | 87 ± 10 | nd | 92 ± 5 | nd | 83 ± 2 | nd | 91 ± 2 | nd |
| **EE2** | 91 ± 10 | nd | 93 ± 14 | nd | 81 ± 3 | nd | 97 ± 4 | nd |
| **E1** | 82 ± 4 | nd | 85 ± 4 | nd | 88 ± 6 | nd | 96 ± 6 | nd |
| **TCS** | 89 ± 3 | nd | 95 ± 2 | nd | 91 ± 1 | nd | 91 ± 1 | nd |

*nd: not determined

**Poor stability of this compound was observed.

**Tab. S4** Matrix effect obtained using the three PES membrane of 0.1 µm pore size (P01, H01 and S01) and PES membrane with 0.45 µm pore size (P045). Green cells highlight 80% ≤ ME% ≤ 120%, yellow cells highlight 50% ≤ ME% < 80% and 120% < ME% ≤ 150%, and red cells highlight ME% < 50% and ME% > 150%.

|  | **ME%** | | | |
| --- | --- | --- | --- | --- |
|  | **P01** | **P045** | **H01** | **S01** |
| **ACS** | 92 | 87 | 85 | 92 |
| **TAU** | 68 | 59 | 49 | 59 |
| **OMT** | 53 | 77 | 83 | 14 |
| **TBR** | 74 | 56 | 40 | 20 |
| **THEOP** | 67 | 75 | 31 | 22 |
| **DMNZ** | 63 | 57 | 60 | 38 |
| **SCL** | 82 | 91 | 76 | 94 |
| **CAFF** | 67 | 62 | 33 | 27 |
| **PFOA** | 122 | 114 | 109 | 111 |
| **HCTZ** | 67 | 78 | 87 | 83 |
| **2,4-D** | 103 | 99 | 99 | 99 |
| **FRSM** | 104 | 102 | 97 | 96 |
| **CMPH** | 105 | 102 | 100 | 104 |
| **CRB** | 96 | 100 | 103 | 103 |
| **KET** | 86 | 102 | 109 | 113 |
| **NAP** | 121 | 124 | 106 | 114 |
| **MTF** | 102 | 103 | 100 | 104 |
| **ATN** | 97 | 96 | 96 | 103 |
| **SALBU** | 106 | 103 | 102 | 108 |
| **TRBT** | 107 | 100 | 99 | 104 |
| **CMQ** | 113 | 117 | 115 | 117 |
| **DIC** | 109 | 99 | 99 | 94 |
| **IBU** | 101 | 102 | 100 | 101 |
| **BP-3** | 90 | 93 | 95 | 101 |
| **GEM** | 112 | 102 | 101 | 108 |
| **FXP** | 66 | 118 | 103 | 137 |
| **MTP** | 96 | 104 | 103 | 111 |
| **CLNB** | 100 | 101 | 102 | 106 |
| **OD-PABA** | 95 | 101 | 100 | 106 |
| **EHMC** | 98 | 98 | 98 | 97 |
| **OC** | 22 | 53 | 55 | 6 |
| **BPA** | 108 | 100 | 94 | 105 |
| **E2** | 94 | 86 | 98 | 104 |
| **EE2** | 91 | 93 | 103 | 95 |
| **E1** | 102 | 96 | 97 | 96 |
| **TCS** | 105 | 98 | 103 | 102 |

**Tab. S5** Results of the stability (S%) in water of the control beaker for K_PESw_ evaluation using the S1 and S2 setup.

|  | **S % (S1 setup)** | **S % (S2 setup)** |
| --- | --- | --- |
| **ACS** | 106 | 120 |
| **THEOP** | 97 | 110 |
| **OMT** | 72 | 96 |
| **DMNZ** | 0 | 0 |
| **CAFF** | 107 | 103 |
| **PFOA** | 92 | 116 |
| **SCL** | 98 | 99 |
| **HCTZ** | 59 | 69 |
| **2,4-D** | 102 | 99 |
| **FRSM** | 105 | 102 |
| **CMPH** | 102 | 98 |
| **CRB** | 103 | 101 |
| **KET** | 91 | 59 |
| **NAP** | 83 | 89 |
| **CMQ** | nd | 92 |
| **MTF** | 103 | 86 |
| **ATN** | 101 | 95 |
| **SLBT** | 95 | 96 |
| **TRBT** | 99 | 96 |
| **DIC** | 110 | 95 |
| **IBU** | 75 | 53 |
| **GEM** | 106 | 100 |
| **BP-3** | 104 | 105 |
| **MTP** | 101 | 102 |
| **OD-PABA** | 38 | 12 |
| **EHMC** | 47 | 4 |
| **CLNB** | 100 | 102 |
| **OC** | 84 | 58 |
| **BPA** | 98 | 116 |
| **E2** | 106 | 157 |
| **EE2** | 115 | 109 |
| **E1** | 94 | 136 |
| **TCS** | 98 | 107 |

**Tab. S6** Results of the partition coefficients (K_PESw_) obtained using the S1 setup for three PES membrane of 0.1 µm pore size of different suppliers (P01, H01 and S01) and PES membrane with 0.45 µm pore size (P045).

|  | **P01** | | **P045** | | **H01** | | **S01** | |
| --- | --- | --- | --- | --- | --- | --- | --- | --- |
|  | K_PESw_ (L/kg) | Log K_PESw_ | K_PESw_ (L/kg) | Log K_PESw_ | K_PESw_ (L/kg) | Log K_PESw_ | K_PESw_ (L/kg) | Log K_PESw_ |
| 2,4-D | 130 ± 8 | 2.11 ± 0.05 | 59 ± 5 | 1.77 ± 0.09 | 103 ± 5 | 2.01 ± 0.04 | 81 ± 4 | 1.86 ± 0.09 |
| CRB | 194 ± 8 | 2.29 ± 0.04 | nd | nd | nd | nd | 63 ± 2 | 1.8 ± 0.03 |
| DIC | 558 ± 29 | 2.69 ± 0.04 | 332 ± 20 | 2.52 ± 0.06 | 257 ± 19 | 2.36 ± 0.07 | 650 ± 27 | 2.81 ± 0.04 |
| IBU | 582 ± 55 | 2.55 ± 0.06 | 164 ± 32 | 2.2 ± 0.2 | nd | nd | 240 ± 18 | 2.38 ± 0.07 |
| BP-3 | 349542 ± 23584 | 5.54 ± 0.07 | 42539 ± 179 | 4.629 ± 0.004 | 100230 ± 3044 | 5.00 ± 0.03 | 91670 ± 836 | 4.962 ± 0.009 |
| GEM | 1996 ± 73 | 3.19 ± 0.03 | 525 ± 16 | 2.72 ± 0.03 | 372 ± 10 | 2.513 ± 0.006 | 1100 ± 17 | 3.041 ± 0.02 |
| MTP | 89 ± 3 | 1.95 ± 0.03 | 53 ± 2 | 1.72 ± 0.04 | 108 ± 6 | 2.01 ± 0.06 | nd | nd |
| OD-PABA | 1912037 ± 62953 | 6.26 ± 0.03 | 53298 ± 777 | 4.73 ± 0.01 | 226805 ± 3685 | 5.35 ± 0.06 | 202887 ± 3003 | 5.307 ± 0.007 |
| EHMC | nd | nd | 108467 ± 823 | 5.035 ± 0.008 | 635228 ± 46812 | 5.8 ± 0.1 | 342923 ± 2531 | 5.535 ± 0.006 |
| CLBT | 219 ± 4 | 2.34 ± 0.02 | 134 ± 0 | 2.127 ± 0.001 | 362 ± 3 | 2.53 ± 0.01 | nd | nd |
| OC | 449783 ± 22841 | 5.65 ± 0.06 | 272479 ± 6814 | 5.44 ± 0.03 | 150384 ± 12917 | 5.15 ± 0.06 | 535330 ± 27424 | 5.73 ± 0.05 |
| BPA | 6534 ± 231 | 3.81 ± 0.04 | 1146 ± 117 | 3.1 ± 0.1 | 1803 ± 47 | 3.26 ± 0.03 | 3624 ± 186 | 3.56 ± 0.06 |
| E2 | 2826 ± 225 | 3.45 ± 0.08 | 519 ± 218 | 2.7 ± 0.4 | 942 ± 230 | 3 ± 0.2 | 1435 ± 225 | 3.2 ± 0.2 |
| EE2 | 6473 ± 340 | 3.81 ± 0.05 | 1300 ± 150 | 3.1 ± 0.1 | 1921 ± 353 | 3.3 ± 0.2 | 4120 ± 431 | 3.6 ± 0.1 |
| E1 | 4713 ± 201 | 3.67 ± 0.04 | 922 ± 85 | 2.96 ± 0.09 | 1382 ± 206 | 3.1 ± 0.1 | 2454 ± 62 | 3.39 ± 0.03 |
| TCS | 218262 ± 5392 | 5.26 ± 0.04 | 59223 ± 893 | 4.77 ± 0.02 | 98218 ± 1124 | 4.99 ± 0.01 | 116152 ± 3928 | 5.06 ± 0.03 |

**Tab. S7** Results of the partition coefficients (Log K_PESw_) obtained using the S1 setup and S2 setup using Pall membranes of 0.1 µm pore size (P01) and extrapolated from the curve of the co-solvent method.

|  | **S1 setup** | **S2 setup** | **Cosolvent** |
| --- | --- | --- | --- |
|  | **Log K_PESw_** | **Log K_PESw_** | **Log K_PESw_** |
| **ACS** | nd | -0.1 ± 0.2 | nd |
| **THEOP** | nd | 2.20 ± 0.06 | 2.13 ± 0.07 |
| **CAFF** | nd | 2.26 ± 0.03 | nd |
| **PFOA** | nd | 1.46 ± 0.03 | 1.14 ± 0.07 |
| **SCL** | nd | 0.5 ± 0.2 | nd |
| **HCTZ** | nd | 2.3 ± 0.1 | 2.23 ± 0.02 |
| **2,4-D** | 2.11 ± 0.05 | 1.86 ± 0.02 | nd |
| **FRSM** | nd | 2.2 ± 0.1 | nd |
| **CMPH** | nd | 2.37 ± 0.07 | 2.35 ± 0.02 |
| **CRB** | 2.29 ± 0.04 | 2.703 ± 0.003 | 2.76 ± 0.02 |
| **KET** | nd | 3.04 ± 0.25 | nd |
| **NAP** | nd | 3.3 ± 0.4 | nd |
| **MTF** | nd | 0.96 ± 0.02 | 1.08 ± 0.05 |
| **ATN** | nd | 1.09 ± 0.07 | nd |
| **SLBT** | nd | 1.11 ± 0.05 | nd |
| **TRBT** | nd | 1.49 ± 0.03 | nd |
| **DCF** | 2.69 ± 0.04 | 3.2 ± 0.6 | nd |
| **IBU** | 2.55 ± 0.06 | 3.2 ± 1.1 | nd |
| **BP-3** | 5.54 ± 0.07 | nd | nd |
| **GEM** | 3.19 ± 0.03 | 3.9 ± 0.8 | nd |
| **MTP** | 1.95 ± 0.03 | 2.10 ± 0.01 | 2.17 ± 0.07 |
| **OD-PABA** | 6.26 ± 0.03 | nd | nd |
| **CLNB** | 2.34 ± 0.02 | 2.5 ± 0.1 | 2.54 ± 0.07 |
| **OC** | 5.65 ± 0.06 | nd | nd |
| **BPA** | 3.81 ± 0.04 | nd | 4.6 ± 0.1 |
| **E2** | 3.45 ± 0.08 | nd | 4.0 ± 0.3 |
| **EE2** | 3.81 ± 0.05 | nd | nd |
| **E1** | 3.67 ± 0.04 | nd | 4.33 ± 0.09 |
| **TCS** | 5.26 ± 0.04 | nd | nd |

***
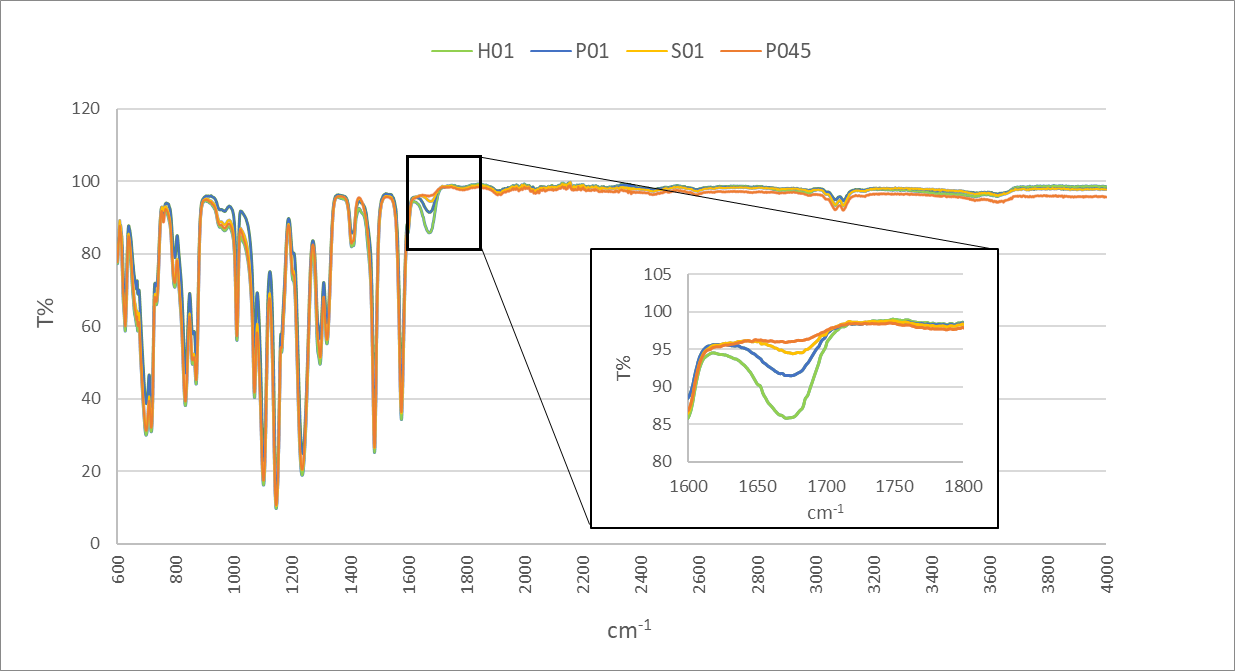
*Fig. S1** ATR-FTIR spectra of the four studied membranes: H01 (green), P01 (blue), S01 (yellow) and P045 (orange).


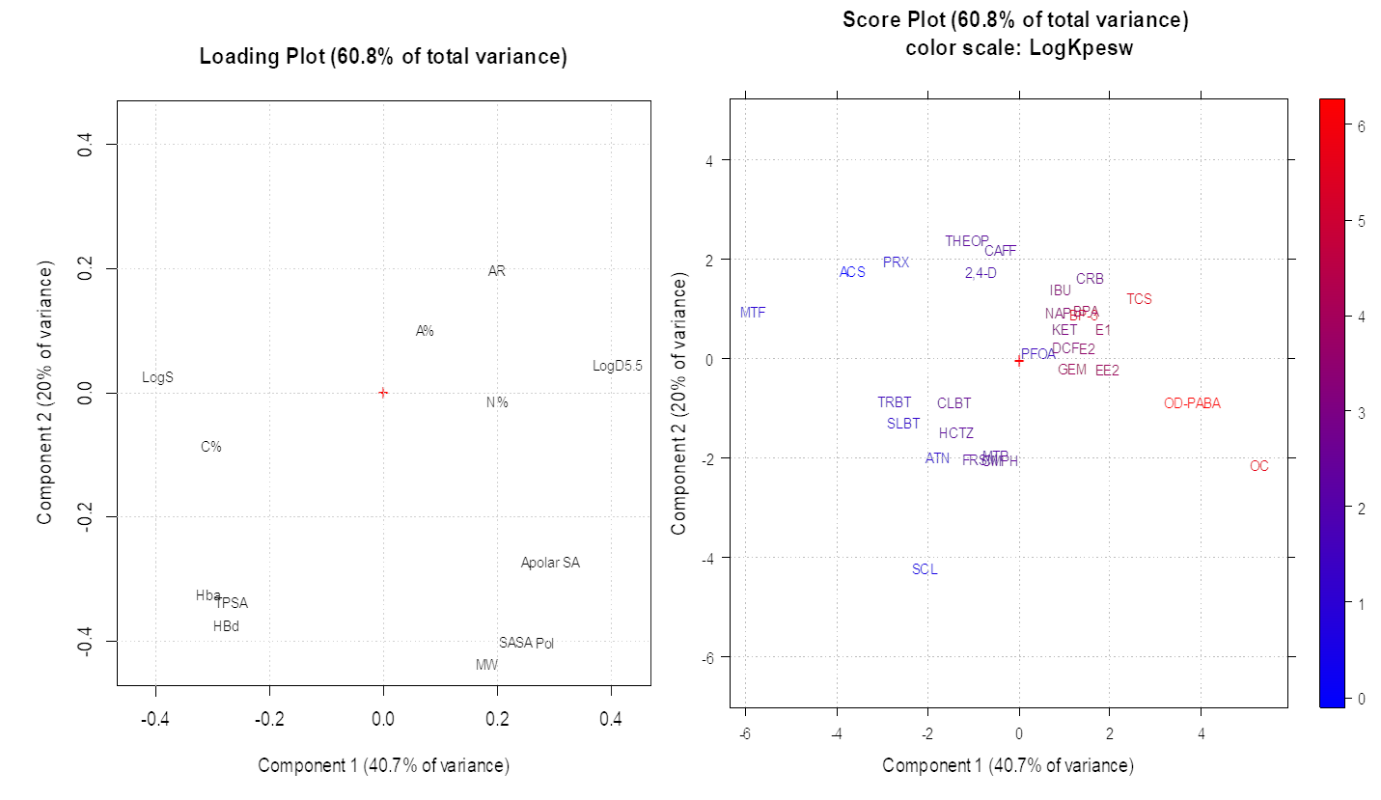


**Fig. S2** PCA results: loading plot (left) and score plot (right). The variable considered are: LogD (pH 5.5) (LogD5.5), Molecular Weight (MW), Number of H-bonding donor groups (Hbd), Number of H-bonding acceptor groups (Hba), Topological Polar Surface Area (TPSA), Number of Aromatic Rings (AR), Polarizability (Pol), Solvent Accessible Surface Area (SASA), Apolar Surface Area (Apolar SA), Solubility (LogS), percentage of neutral analyte pH = 5.5 (N%), percentage of anionic analyte pH = 5.5 (A%), percentage of cationic analyte pH = 5.5 (C%). The colour vector in the score plot present the values of LogK_PESw_ associated to the objects (lower value in blue and higher in red).
